# Balancing act: persistence of the red fox in the dog-dominated landscapes of the Trans-Himalaya

**DOI:** 10.1098/rspb.2025.0333

**Published:** 2025-06-04

**Authors:** Herman Ramesh, Rumaan Malhotra, Sai Khurana, Deepraj Pandey, Tandup Chhering, Kulbhushansingh Ramesh Suryawanshi

**Affiliations:** ^1^ Wildlife Biology and Conservation Program, National Centre for Biological Sciences, Tata Institute of Fundamental Research, Bangalore, Karnataka 560065, India; ^2^ High Altitude Program, Nature Conservation Foundation, Mysuru, Karnataka 570017, India; ^3^ Makerspace, Mphasis AI & Applied Tech Lab, Ashoka University, Sonipat, Haryana 131029, India; ^4^ Snow Leopard Trust, Seattle, WA 98103, USA; ^5^ Future Flourishing Program, CIFAR, Toronto, Ontario M5G 1M1, Canada

**Keywords:** *Vulpes vulpes*, vigilance, intraguild predation, coexistence, dogs, sympatry

## Abstract

Free-ranging dogs pose a growing threat to wildlife globally. In the Indian Trans-Himalaya, growing populations of dogs raise concerns about their impact on native carnivores. Red foxes in Spiti Valley share spatial and dietary niches with dogs, despite intraguild killing pressure. Sampling across a gradient of dog density in the winter, we investigated two potential mechanisms that might enable the observed sympatry between foxes and dogs. Using a cue-based foraging station experiment, we investigated the use of anti-predatory vigilance by foxes. We also used a camera trap array to assess temporal partitioning between the two canids and the relative abundance of foxes across the dog-density gradient. Foxes only increased vigilance in response to simulated dog presence at sites with high dog densities. We found that temporal overlap was low across the dog-density gradient in the winter. Fox relative abundances increased with dog-density, indicating a potential lack of apparent top-down effects by dogs. Our findings suggest that while temporal partitioning may reduce encounters, increased vigilance in high-dog-density areas may be crucial for coexistence. We highlight the complex behavioural mechanisms facilitating the persistence of a generalist mesocarnivore in the face of increasing pressure from free-ranging dogs and underscore the need for similar investigations in other human-dominated landscapes.

## 1. Introduction

The expansion of the anthropogenic footprint has resulted in the spread of human-associated carnivores such as cats and dogs [1,2]. Their use of human food subsidies enables them to persist at higher densities than native carnivores [3]. They can have disproportionate impacts on native carnivores as predators and competitors [4]. Increasing numbers of cats and dogs are accompanied by altered community structures, behaviours and extinction or extirpation of native species [1,5,6]. While the negative impacts of human-associated carnivores on islands and the global north are well established, research into how these species interact with, and affect communities in biodiverse rich, global south regions is lacking [1,7–10]. While the impact of cats on faunal communities is well documented, dogs have received less attention, despite being more abundant and widespread [1,11–13].

Direct interactions between carnivores are largely antagonistic and favour the larger animal. In extreme cases, such antagonistic interactions can result in intraguild killing or predation [14]. Subordinate carnivores often reduce the chances of encountering a dominant carnivore in a landscape through spatial or temporal segregation. This would mean reducing or ceasing the use of certain areas or times of day where the risk of encountering a dominant predator is high [15–17]. When encounters do take place, animals avoid negative outcomes by engaging in anti-predatory behaviours which reduce the chances of capture through fine-scale spatiotemporal avoidance [18–20]. Vigilance allows animals to collect information on and potentially avoid approaching threats, especially when there is spatiotemporal overlap with predators [21–23]. For example, red foxes display higher vigilance when using high-risk carrion sites where they spatiotemporally overlap with intraguild predators [24]. Similarly, vigilance behaviour is thought to enable sympatry between pine martens and red foxes despite spatiotemporal overlap in Sweden [25]. Engaging in vigilance can allow animals to avoid threats at finer time scales, albeit while reducing time allocated to other activities like foraging or prey handling [18,26].

In recent years, regions from across the Indian Trans-Himalaya have reported a rise in the numbers of free-ranging dogs [27–30]. They are a major conservation concern, with densities reaching 300 individuals per 100 km^2^ in some regions [29]. In some regions, dog attacks even result in human mortality [30]. Spiti Valley, in the Indian state of Himachal Pradesh, is one such region [27,31,32], where a recent rise in free-ranging dog numbers is accompanied by reports of them hunting wild ungulates, hybridizing with wolves, and killing more livestock than the apex predators of the region [33,34]. Their antagonistic interactions with the prevalent mesocarnivore, the red fox (*Vulpes vulpes*), in Spiti are well recorded, with several cases of intraguild killing being reported [31].

Red foxes and wolves (*Canis lupus*) are the two native canids in Spiti. Wolves in Spiti occupy large territories in low densities, and often hunt livestock, leading to conflict with local herders [35]. While wolves persist in the landscape, their low densities and large home ranges mean encounters are rare [36]. Foxes in Spiti are opportunistic in their habits, consuming small mammals, plants and fruits, insects, livestock and ungulate carrion, and human-derived food [32,37,38]. They frequently use anthropogenic spaces such as villages (as seen in several red fox populations globally) and often exploit human food subsidies through livestock carcasses and garbage dumps [32,37,38]. Their presence in these villages, and their use of human-subsidized food brings them into direct contact and conflict with dogs, which reside in and around villages and heavily depend on human food subsidies [12,31,32,38]. In the harsh winter months, foxes in Spiti depend on human-subsidized food sources such as domestic garbage and livestock carrion and spatially cluster around villages and roads [32,39]. Studies show that despite sharing close space and diet with an intraguild predator, fox and dog presence in Spiti is positively correlated around human settlements [32]. Fox occupancy in Spiti is highest in areas closer to roads, given the presence of dogs in the landscape [39].

We aimed to understand the mechanisms that may enable the apparent sympatry between a mesocarnivore, the red fox and an intraguild predator, free-ranging dogs in a resource-sparse landscape. Spiti represented an ideal system to understand how foxes may respond to the presence of free-ranging dogs. Not only do foxes and dogs here share spatial, dietary and temporal niches, but dog abundances in Spiti also form a natural gradient, with some villages hosting more dogs than others [27,32,38,39]. This dog-density gradient represents a gradient of intraguild predation risk for foxes. It also represents a gradient of interspecific competitor presence for foxes accessing human-derived food. Moreover, our study period in the harsh winters of Spiti offered an opportunity to collect responses from animals during a period when resources are scarce, and niche overlaps may increase, leading to increased encounters [40].

While spatial and dietary niches of the two canids in Spiti may overlap, foxes show some temporal partitioning of peak activity times from dogs (temporal overlap, Δ = 30%) in the summer (May to October) [39]. However, resource-scarce periods (like a snowy winter) can lead to higher temporal activity overlaps between animals exploiting similar resources [40,41]. If encounters do take place, anti-predator behaviours like vigilance, which can mitigate the risk of getting captured and killed, become key to continued survival [14,19,20,26]. Subordinate predators thus engage in vigilant behaviour when the perceived risk of encountering a predator is high [18,25].

Using simulated dog presence cues at foraging stations, we investigated the use of vigilance as a potential mechanism that allows foxes to use resource-rich villages, despite niche overlaps with an intraguild predator. By setting up these cue-based foraging stations across a gradient of dog-density, we sought to understand if foxes modulate anti-predatory behaviours such as vigilance, when threat from dogs is greater. While studies exploring the role of spatial complexity, predator type, and predator presence on vigilance exist, there has been no examination of how predator density affects anti-predatory behaviour and vigilance in animals and carnivores [25,42–44].

Due to the potential risk of mortality that encountering dogs imposes, we expected foxes across our gradient to increase vigilance under simulated dog presence. We expected the elicited vigilance response to be the greatest at high dog-density sites [45].

While a previous study found that foxes and dogs show some temporal partitioning (Δ = 30%) in Spiti in the summer months at broad spatial scales, we sought to understand if the same persists in the resource-sparse winter, at finer scales around villages [39]. We also aimed to describe how the diel activity patterns of the two canids changes across the gradient of dog density. We deployed a camera trap array around human-derived food sources (garbage and carcass dumps) and trails with fox signs within villages that fell into the dog-density gradient. To understand how fox presence is affected by the density of free-ranging dogs, we also used a subset of our camera trap array to estimate the relative abundance of foxes across a dog-density gradient.

## 2. Methods

### (a) Study location

**Figure 1.**
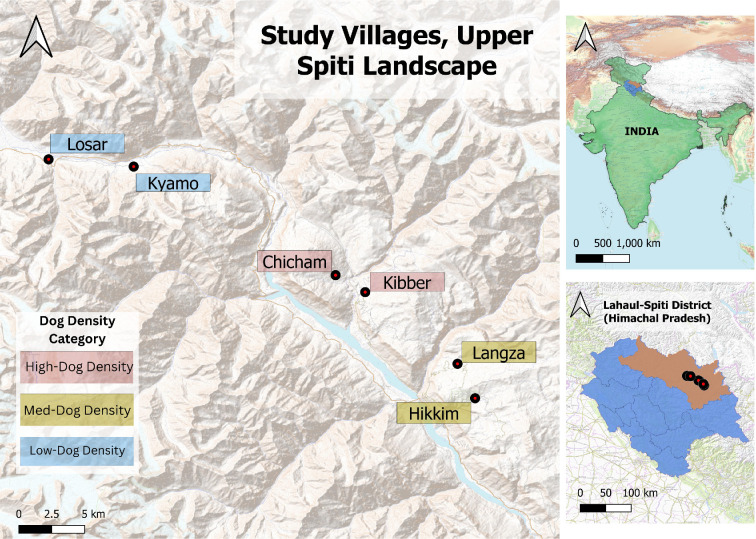
Locations of study villages within Spiti Valley, Himachal Pradesh, India.

Our study area was the Upper Spiti Landscape, which is situated in Himachal Pradesh’s Lahaul-Spiti district (between 32°10′0″ N, 77°40′0″ E and 32°40′0″ N, 77°40′0″ E) (figure 1). It’s a part of the Indian Trans-Himalaya and falls within the rain shadow of the Pir Panjal range. The region is arid, with precipitation mainly coming in the form of snow [27]. The landscape is primarily composed of alpine steppe and is rugged, with elevations between 3600 m and 6700 m [46,47]. Temperatures in the summer (May to October) can rise to highs of 30°C, while dipping to almost −40°C in the winter [34,47].

Wolves and red foxes are the two native canids in the landscape. Snow leopards (*Panthera uncia*), along with wolves, represent the apex predators of the community. The other major mammalian fauna includes ibex (*Capra sibirica*), blue sheep (*Pseudois nayaur*), stone martens (*Martes foina*), pale weasels (*Mustela altaica*) and pika (*Ochotona roylei*) [48].

Most residents practice agropastoralism, with barley, black pea and green pea being the primary crops. Livestock holdings primarily consist of cattle-yak hybrids, yak, sheep, donkeys and horses, while some villages also have smallholdings of goats [49]. Tourism is an important source of income in the winter months, when some villages become hotspots for snow leopard sightings [50].

### (b) Establishing the dog-density gradient

We censused dog populations across 30 villages within the Spiti subdivision in December 2023, using a modified double observer framework [27,51]. We systematically partitioned the areas within and around each village into multiple trails (at least two in each village). These trails were laid out to ensure maximum coverage and visibility of the residential and agricultural lands of the villages. The length of each trail varied between 150 m and 350 m. Teams with two observers each would simultaneously walk each trail, independently enumerating all dogs in that section of the village. Upon completion, the teams would exchange trails and repeat the process. Each trail was thus walked twice. The mean dog consensus counts for each trail were tallied to obtain the total dog numbers for a village. Both replicates for each trail would be completed in the same day, within 1.5 h of each other. Owing to the small sizes of villages in Spiti, the lack of vegetation and the conspicuous, large size of free-ranging dogs, this method allowed us to obtain a census count for each settlement, while minimizing observer bias.

We selected six villages which we categorized into high (Kibber and Chicham), medium (Langza and Hikkim) and low (Kyamo and Losar) based on the relative dog densities within each (see electronic supplementary material; figure 1). We also ensured to select villages with at least 20 households, since dog and fox presence is positively correlated with household number and food availability [7,12,13]. Our low-dog-density villages had dog densities between 2 and 4 dogs per km^2^, medium dog densities were between 10 and 15 dogs per km^2^ and high-density villages were those with more than 30 dogs per km^2^. We repeated our dog census in these selected villages again in March 2024, to ensure that the measured dog densities had not changed.

### (c) Measuring fox vigilance

Since our broad goal was to understand how foxes living around villages minimize interactions with dogs, we limited our enquiries to areas within a 1 km radius from the centre of each village. We chose this distance since we were certain that both dogs and foxes were active within this area [52,53].

We set up cue-based foraging stations, on the outskirts of our study villages (300−900m away from centre). These were locations which had been used by foxes recently but lacked signs of recent dog activity. This was done to decrease the chances of dogs depleting our foraging stations before the arrival of a fox, which would result in the failure of the experiment. Since the spatial structure of a location can affect risk perception in animals, we avoided very rugged areas in favour of relatively flatter pastures [42].

We collected vigilance responses from foxes under two paradigms of simulated dog presence. We simulated the imminent presence of dogs at a location using automated behavioural response units (hereafter ABR units), which played a sequence of barks when an animal was detected. Our ABR units consisted of an independent motion sensor module and a speaker unit (see electronic supplementary material). We also simulated the past presence of dogs at these locations using fresh dog scat. We also included control treatments from a non-threatening animal that is common in the landscape, cows (dung and recorded moos) [54]. The vigilance response elicited by these paired control cues would be crucial to rule out neophobia as a confound in our experiment [54]. Dung acts as a positive control for scat in our experiment, but since our study locations were within pastures close to villages, cow dung was a common occurrence across our study landscape.

A total of six foraging stations were set up at selected locations in our study villages (one foraging station per village). A foraging station consisted of a feeding tray (large flat rock), a Browning Strike Force trail camera (Browning, Utah, USA) and an ABR unit. We baited the foraging stations with one boiled egg, for each replicate, which was mashed onto the feeding tray to ensure that a fox had to stay in the camera’s view to deplete the food. Our cameras were set to record 20 s videos on trigger, with a 1 s delay between each video. We ran the foraging station experiment from 15 February 2024 to 30 April 2024. Between this period, the experiment was performed simultaneously in all six villages.

**Figure 2.**
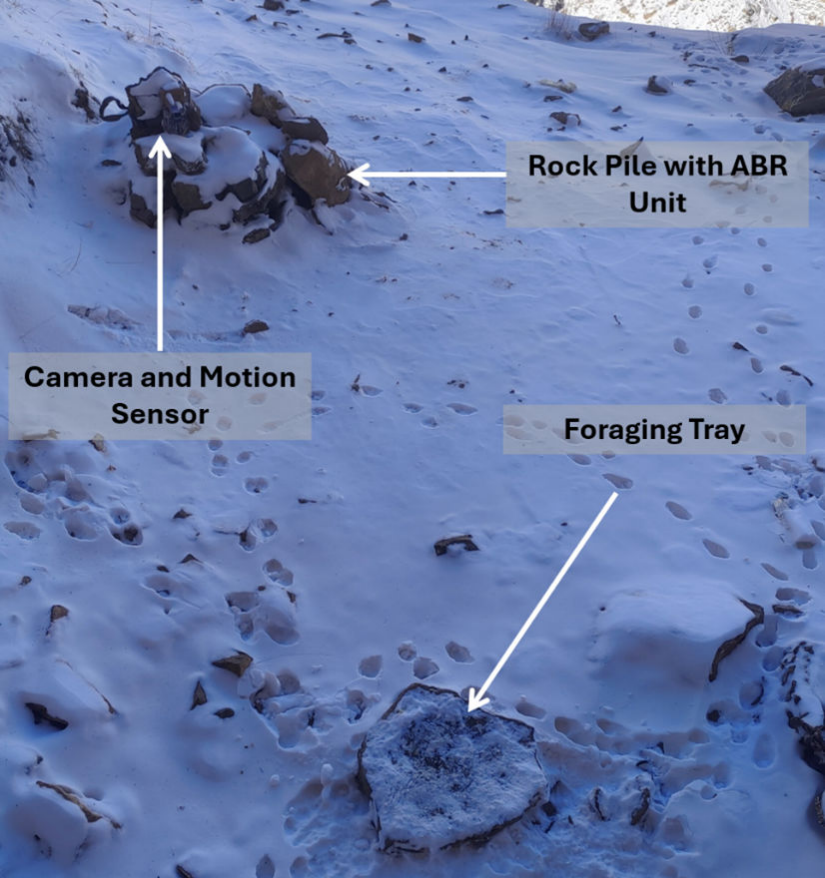
Layout of a foraging station at Kibber village.

The stations were set up at selected locations, devoid of obstructions and with no signs of recent dog activity. We tethered our cameras to rock piles, within which our ABR units were concealed. The motion sensor module was positioned such that its field of view was smaller than, and fully contained within, the camera’s field of view. The foraging trays were placed 2–3 m away from the camera (figure 2). The foraging stations were set up such that they were framed in the centre of the recording frame of the camera. Before the commencement of the experiment, we baited the foraging stations without any treatment for two nights to habituate foxes to feed at our stations. We ran our experiment for a maximum of four nights in a week, with treatments being changed between nights.

We collected fresh dog scat and cow dung [1,4,5] hours before commencement of experiment). The area around the foraging station (10–15m) was cleared of any old dog scat or cow dung, following which, we placed the collected dung/scat within 5 cm of the foraging tray. We loaded our ABR units with 5 s sequences from dogs (barking) and cows (moos). The ABR units were programmed to play the sounds 5 s after an animal was detected. This was followed by a 5 min quiet period, when the ABR would not re-trigger. A single exemplar of barks and moos sequences was used.

We baited our foraging stations and applied our treatments in the late afternoon (between 14.00 and 17.00 local time) across all sites. Fox vigilance was measured as the proportion of time foxes spent with their heads up and looking around. A ‘visit’ by a fox started once a fox had discovered and started feeding on the bait. Since our stations may receive visits from multiple foxes, or multiple visits from an individual in a night, we defined visits as fox detections separated by at least 30 min. We only measured vigilance responses from the first visit of a fox at the foraging stations, as subsequent visitors may find the foraging stations depleted of food.

Since our foraging trays were at ground level, feeding required foxes to look down. Vigilant behaviour was defined as a clear raising of the head (with or without mastication of food in the mouth) to look around (see supplement). We measured the time foxes spent in such behaviour as a proportion of the total amount of time they spent on the camera and feeding. Since there may be multiple fox visits in a night (by the same or different individuals), the proportion of time spent looking was calculated only from the first visit of a fox (when food was not depleted). This was to ensure that we only collected vigilance responses from foxes presented with a consistent amount of food across replicates. We ensured consistency between replicates by discarding any observations where the camera failed to record the first visit entirely, if multiple foxes visit the station simultaneously, if the ABR unit failed to trigger, or if the bait was consumed before the arrival of a fox.

We fit a beta regression model with a logit link to understand how our treatment dog cues affected fox vigilance across our dog-density categories. A beta regression was deemed suitable as it is well-suited for proportion data (such as vigilance time) and addresses deviations from normality and homoscedasticity [55]. The model included individual treatment type (barks, moos, scat or dung), dog-density category and their interaction as predictors. The inclusion of the interaction term allowed us to evaluate how the effects of dog cues on vigilance differed across dog-density levels. We set the low dog-density and dung treatments as the reference categories for the model outputs. We fit the beta model using the *betareg* package in R [56].

We also performed pairwise comparisons of the vigilance elicited by each treatment along with its paired control within each dog-density category using estimated marginal means (EMMs) (or least square means) derived from the model, calculated with the *emmeans* package in R [57]. EMMs provide adjusted predictions for each treatment, accounting for other variables in the model, and allow for pairwise comparison of treatments with their controls.

### (d) Measuring fox relative abundance

For each village, we chose two trails that had fox signs. Reconyx Hyperfire 2 cameras were placed 30 cm above ground level and tethered to a stone tower constructed by piling stones, or to human-built structures if available. The cameras were set to capture a series of five images in quick succession, followed by a 5 s quiet period.

We used the daily encounter rate of foxes within our study areas as a proxy for relative abundance [58,59]. We included fox detections from a 31 days period between 15 March and 15 April 2024. This was the longest period during which the two trail cameras in each village were active and devoid of snow obstructions. While we acknowledge that a measure of relative abundance that is solely based on encounter rates may be misinformed in certain cases, it serves as a simple but useful metric to compare the relative presence of highly active, but individually unmarked animals such as foxes.

To assess the robustness of these counts and quantify uncertainty, we implemented a nonparametric bootstrap procedure. Specifically, we resampled our fox detection data 10 000 times with replacement. For each resample, we computed the total number of fox detections within each dog-density category. This generated a distribution of counts for each category from which we derived mean estimates and 95% confidence intervals. To statistically compare fox relative abundance among the dog-density categories, we performed pairwise Z tests using the bootstrap replicates (with Bonferroni adjustment).

For all analyses, we only included consecutive species detections in our data if they were separated by at least 30 min at any camera location [59].

### (e) Measuring fox and dog temporal activity

To measure and compare the temporal activity of foxes and dogs, we identified dump sites (carcasses and/or garbage) and trails that had recently been used by foxes through foot surveys. A temporal subset of the data from these trail cameras was used to estimate the relative abundance index mentioned in §2d. We chose to include garbage and carcass dumps since they represent an important winter food resource for Himalayan foxes and dogs in the region, and are thus, a location for potential interference competition and regular activity [32,37,38]. We placed 2–4 Reconyx Hyperfire 2 (Reconyx, Wisconsin, USA) cameras at the identified garbage and carcass dump sites at each village. The cameras were set to capture a series of five images in quick succession, followed by a 5 s quiet period. To measure fox and dog activity, we deployed 29 cameras, distributed across our 6 sites over a period of 5 months (December 2023 to April 2024).

We used kernel density estimates, a non-parametric method which estimates the probability density of an animal occurring at a time within the 24 h daily cycle to visualize and describe the activity patterns of dogs and foxes within our categorized sites [60,61]. We calculated the overlap coefficient (Δ) to quantify the amount of temporal overlap between the two species across our dog-density categories [62]. Since we had sufficient detections across all our dog categories for both species, we chose to use the Δ_4_ non-parametric estimator to arrive at our overlap coefficient [63]. We quantified the uncertainty around these estimates by non-parametric bootstrapping (1000 iterations), through which we obtained 95% confidence intervals (CI) around our overlap coefficient estimates for each dog category. We used the *bootstrap* function of the *Overlap* package in R for estimating confidence intervals around our temporal overlap estimates [64].

We performed pairwise comparisons of dog activity and fox activity across our dog-density categories using the *compareCkern* function from the *Activity* package in R [65]. We wanted to understand if foxes and dogs changed their use of time when dog-densities change. The function compares the observed overlap between two circular distributions with a randomized null distribution and determines the probability of the observed overlap arising by chance.

## 3. Results

### (a) Fox vigilance

We ran our foraging station experiment successfully on 158 nights across all our sites, resulting in 24.3 h of first-visit fox footage. Across the 158 runs of the experiment, foxes continued feeding until the foraging station was depleted, and did not flee, regardless of the supplied cue. We often had multiple visits by foxes (separated by 30 min) at our foraging stations, but we only enumerated the proportion of time spent being vigilant from the first fox that visited each night.

#### (i) Low-dog-density sites

**Figure 3.**
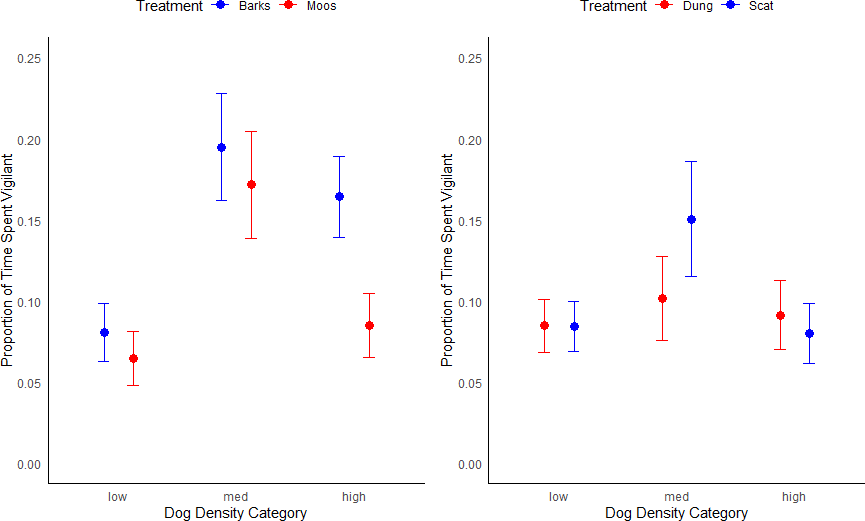
(Left) The proportion of time that foxes spent being vigilant across the dog-density gradient, under simulated current presence of dogs (dog barks through ABR) and its paired positive control (cow moos through ABR). (Right) The proportion of time that foxes spent being vigilant across the dog-density gradient, under simulated recent presence of dogs (using fresh scat) and its paired positive control (fresh cow dung).

At low-dog-density sites, vigilance remained consistently low across all treatments (figure 3). None of the treatments, barks (β=−0.053, *p* = 0.742), moos (β=−0.294, *p* = 0.093) or scat (β=−0.006, *p* = 0.970), elicited significantly different responses compared with dung. Pairwise comparisons between treatments revealed no significant differences in vigilance among barks, moos, scat or dung (*p* > 0.05 for all contrasts). Foxes at these sites continued feeding without raising their heads more when dog presence is simulated.

#### (ii) Medium-dog-density sites

At medium-dog-density sites, vigilance increased significantly for both barks (β = 0.816, *p* < 0.01) and moos (β = 0.901, *p* < 0.01) compared with dung (figure 3). Scat elicited a smaller increase in fox vigilance (β = 0.457, *p* = 0.067) compared with dung at medium dog density, but this was not statistically significant. Pairwise contrasts between barks and moos showed no significant difference (*p* > 0.05), suggesting that the response to these auditory cues was comparable. While foxes at our medium dog-density sites displayed heightened vigilance upon hearing dog barks, they also showed a similar increase in vigilance upon hearing the control sound. Thus, we cannot reliably attribute the increase in vigilance as a specific response to dog cues.

#### (iii) High-dog-density sites

At high-dog-density sites, vigilance increased significantly in response to barks (β = 0.724, *p* = 0.0015) compared with dung. In contrast, neither moos (β = 0.217, *p* = 0.392) nor scat (β = −0.136, *p* = 0.561) elicited significant differences in vigilance compared with dung at high dog density (figure 3). Pairwise comparisons revealed that vigilance was significantly higher for barks compared with moos (*p* < 0.001) at these sites. These results indicate that foxes at high-dog-density sites probably associate barks with increased risk and respond with increased vigilance compared with a control sound. Here too, dog scat failed to elicit any difference in vigilance (figure 3).

### (b) Fox relative abundance

Between 15 March 2024 and 15 April 2024, our cameras recorded 48 fox detections (95% CI: 38−58) at high-dog-density sites, 43 detections (95% CI: 33−53) at medium-dog-density sites and 20 detections (95% CI: 12−28) at low-dog-density sites. Pairwise comparisons based on our bootstrap-derived means and standard errors indicated significant differences between fox detections at low-density sites versus both medium- (Z = −3.52; *p* < 0.01) and high-density sites (Z = −4.22; *p* < 0.01). In contrast, the difference between medium- and high-dog-density sites was not statistically significant (Z = −0.65; *p* > 0.1).

### (c) Fox and dog activity

The camera trap array was active for 2383 trap-nights, over which we obtained 2140 independent detections of foxes and 2960 detections of dogs across the 6 sampling villages. We also had sporadic detections of other wild carnivores in the area such as wolves (*n* = 72), snow leopards (*n* = 7) and stone martens (*n* = 26) (see supplement). These were filtered from our datasets since our primary interest was to investigate interaction between foxes and dogs.

**Figure 4.**
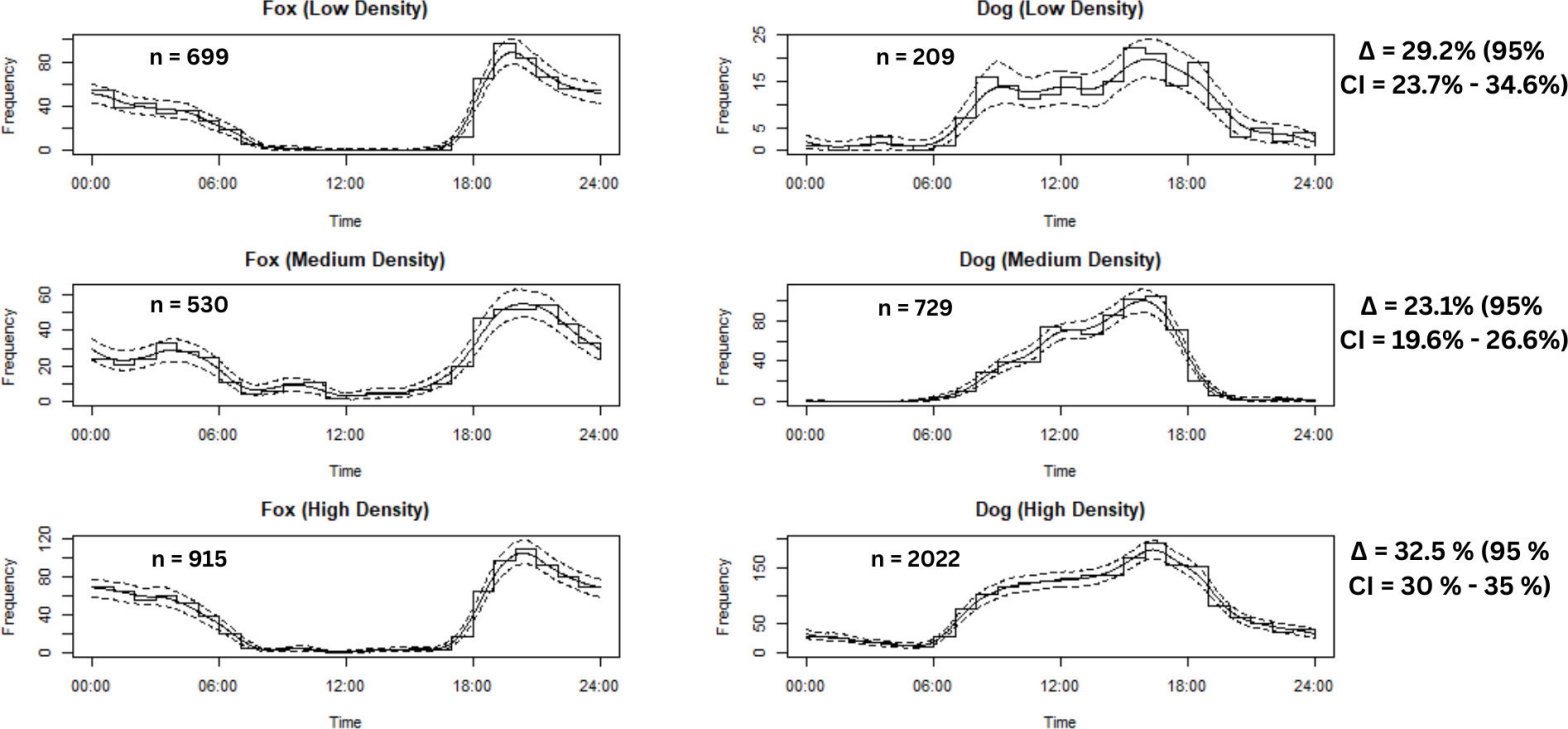
Kernel density estimates of dog and fox diel activity at low-, medium- and high-dog-density sites. Dotted lines represent 95% confidence intervals around the activity estimates.

Our fitted kernel density plots showed that foxes were largely nocturnal, with activity peaking between 18.00 and 00.00 across the dog-density categories (figure 4). While foxes in our low and high dog-density sites showed a unimodal activity pattern, with activity declining after 06.00, foxes in the medium dog-density sites showed a trimodal activity pattern, with two smaller peaks between 00.00 and 12.00. When compared pairwise, foxes at the medium-dog-density sites showed a significantly different activity (*p* < 0.01) than those at high- or low-dog-density sites, which showed similar activity (*p* > 0.1).

Dogs across our sites remained predominantly diurnal, with activity peaks in the afternoon between 12.00 and 18.00 across all sites (figure 4). We had the fewest dog detections at the low dog-density sites, and here, dogs gradually reduce their activity after 18.00, and reach near zero activity between 00.00 and 06.00 (five detections). At the medium dog-density sites, dogs show a very sharp decline in activity after 18.00 h and are totally inactive in the night (between 00.00 and 06.00; one detection). However, at our high-dog-density sites, dogs remained consistently active throughout the 24 h diel period; while dog activity does reduce between 18.00 and 00.00, dogs continue their activity during this time (*n* = 112 detections). Despite the high amount of night-time dog activity at the high-dog-density sites, the activity pattern of dogs here is not statistically different from dogs at low-dog-density sites (*p* > 0.05). Pairwise comparisons of activity revealed that dogs at our medium-dog-density sites show a distinct pattern of activity compared with dogs at low- or high-dog-density sites (*p* < 0.01).

Across our sites, foxes showed activity peaks distinct from those of dogs. Contrary to our expectations, the highest temporal overlap between the two species was seen at the high-dog-density sites (Δ = 32.5% (95% CI = 30%–35 %)), while the lowest overlap was seen at the medium-dog-density sites (Δ = 23.1% (95 % CI = 19.6%–26.6 %)). At the low-density sites, overlap between the two species was 29.2% (95% CI = 23.7%–34.6 %). The low overlap values indicate that fox and free-ranging-dog activity in Spiti is temporally partitioned across the dog-density gradient.

## 4. Discussion

As dog populations increase worldwide, there is an increasing need to understand their impact on wildlife, and the mechanisms through which vulnerable wildlife survive [1,2,13]. Across a dog-density gradient in the Indian Trans-Himalaya, we found that red fox relative abundances may be higher where dog densities are greater [32]. We investigated temporal partitioning and anti-predatory vigilance behaviours as potential mechanisms for the observed sympatry [19,20,26,66–68]. We found that while temporal partitioning between the two canids persists across the dog-density gradient [39], only foxes at high dog densities modulated vigilance in response to simulated dog presence.

A previous study had examined temporal activity of foxes and dogs at broad spatial scales in Spiti in the summer season and found evidence of temporal partitioning between the two [39]. Our study examined temporal activity of the two canids at much finer scales, closer to human habitation (within 1 km of villages), in the resource-sparse winter, and along a gradient of intraguild predator density. Foxes were nocturnal and dogs were diurnal in their activity throughout the dog-density gradient. We found that the patterns of temporal partitioning between foxes and dogs seen in the summer in Spiti are conserved in the winter. Temporal partitioning from larger predators by subordinate carnivores has been seen in numerous systems [15,16,67,69]. Partitioning of activity peaks has also been observed in other systems where foxes and dogs are sympatric [39,68,70]. For example, in areas where Indian foxes (*Vulpes bengalensis*) are sympatric with dogs, their activity peaks when dog activity reduces [70]. The low temporal overlap between the two species (22–32%) that we found across the dog-density gradient could represent avoidance or partitioning by foxes. Alternatively, the activity patterns of foxes in Spiti may be independent of dogs, as the temporal activity we observed is very similar to those reported from other systems. Red foxes are generally crepuscular or nocturnal regardless of hunting pressure, presence of other carnivores, and human disturbances [15,71,72]. Sampling fox responses from areas where dogs are entirely absent may yield further insights into understanding if the fox activity patterns we describe are a shifted response to the presence of dogs (even at low densities), or the inherent pattern of red fox activity in the landscape.

Vigilance, while highly effective at gathering information about approaching threats, can divert time from other crucial activities such as foraging or food handling. It is thus an energetically expensive activity to engage in. For animals living in areas where risk of encountering predators remains high, anti-predatory behaviours like vigilance become crucial in increasing survivorship [20,26]. Although foxes in our high-dog-density sites show temporal partitioning of activity from dogs, nocturnal dog activity here remains high. Thus, temporal avoidance alone may not fully mitigate the risk of encounters with dogs here. Only at these high-dog-density sites, which may also be ‘riskier’ sites, did foxes raise the proportion of time allocated to vigilance. The fact that barking elicits heightened vigilance only at the high-dog-density sites lends further support to the idea that temporal avoidance alone may not be sufficient to avoid dangerous encounters at these ‘riskier’ sites.

Studies across several systems show that while broad-scale spatiotemporal segregation is important in reducing intraguild aggression, mesocarnivores with access to high quality resources may use vigilance and predator recognition to continue exploiting resources in high-risk areas through fine-scale spatiotemporal avoidance [43,44,73–75]. Reactive avoidance like this can allow for continued utilization of shared resources while decreasing chances of antagonistic encounters [43,75,76]. Investigations into fox occurrence in the Indian Trans-Himalaya reveal that fox occurrence is higher where the use of human-subsidized food by foxes is higher, regardless of dog presence [38]. Considering the benefit accorded by human-derived food, foxes in other regions of the Trans-Himalaya may also exploit these food subsidies while avoiding interactions with dogs at fine scales. We obtained several consecutive photo captures of foxes and dogs that were minutes or seconds apart in our high-dog-density sites, signifying avoidance at fine temporal scales. Foxes at such ‘high-risk’ sites that are unable to raise vigilance in response to dog cues may face increased mortality, while those that can raise vigilance may avoid attacks or capture and continue persisting.

The lack of a response to dog cues at low dog-density sites may either indicate a non-recognition of dog cues by foxes here or a lack of fear/perceived risk upon encountering dog cues. Considering the low density of dogs, and the lower relative abundance of foxes, encounters between the species are probably the fewest here compared with other sites. Foxes that encounter dogs less can not only have a lower level of perceived risk from dogs but might not recognize dog cues due to a lack of experience. It is important to consider the possibility that an individual fox may move between dog-density regimes, despite the on-ground snow cover. Although collaring data from Spiti shows that foxes stay within village bounds during the winter [52], incorporating individual animal IDs and integrating telemetry data may reveal specific and nuanced insights into how foxes mitigate risk of encounters with dogs at these low dog-density sites. We also note that our bark playbacks only consisted of sounds from a single individual. Since dogs form packs of varying sizes and encountering a larger pack may be ‘riskier’ for a fox, a chorus of barks may be a more reliable way to simulate ‘high-risk’ imminent presence of dogs at a station.

We believe the lower fox–dog temporal overlap, as well as the constricted dog activity, at the medium-dog-density sites, may be because of the presence of wolves at these sites. While intraguild killing in canids is highly mediated by size, aggression is strongest when the species are closer in body sizes [14,77]. Wolves can exert top-down non-lethal effects on dogs where the two are sympatric [78]. While we only sporadically detected wolves at our low- (*n* = 1) and high-dog-density (*n* = 4) sites, respectively, we detected 67 wolves at the medium-dog-density sites in the study period. Wolf activity was primarily nocturnal (supplement), thus, the sharp decline in nocturnal dog activity could be due to temporal avoidance of wolves (a large apex predator) by dogs, which are smaller than and subordinate to wolves [14,18]. Lower nocturnal activity by dogs, thus manifests as lower dog–fox temporal overlap at medium-dog-density sites.

We could not conclude if the foxes at the medium-dog-density sites were responding to the cues of dogs, or just a novel stimulus in the landscape at our medium-dog-density sites. While foxes at medium-dog-density sites likely encountered dogs the least due to low temporal overlap (Δ = 22.3 %), these sites also had more frequent wolf activity compared with others. The presence of an additional large carnivore along with dogs may lead to foxes experiencing a higher level of perceived risk at these sites, regardless of our cues. While some studies show that wolves can exert top-down effects on foxes [78], others show foxes benefiting in wolf presence through carrion provisioning and suppression of fox predators [79]. Parsing the effects of wolf presence on fox and dog activity and further investigation into the dynamics of the three canids at these medium-dog-density sites may help in understanding how dogs affect the carnivore community and intraguild interactions.

The positive correlation we observed between dog density and fox relative abundance is similar to what others have reported from the region, albeit at a broader scale [32]. This positive correlation might be an indicator that in resource-sparse systems like Spiti, the top-down effects (lethal and non-consumptive) exerted by dogs on foxes, are not enough of a cost to offset the benefit accorded through human-derived food. A previous study from the Indian Trans-Himalaya, which looked at the use of anthropogenic food by foxes, showed that fox presence was higher in areas where they exploited human-derived food, regardless of the presence of dogs [38]. It must also be noted that the higher dog-densities likely correspond to higher food availability in a village. Our findings are from the winter season, when dependence on, and competition for human-derived food is likely high between the two canids. We must note that our relative abundance index is merely a daily encounter rate. Foxes may deal with a higher density of dogs (competitors) and lower availability of resources with increased daily movement, resulting in higher observed relative abundances. Further studies may benefit from quantifying the vital rates (birth and death rates) of foxes across this dog-density gradient more continuously. Demographic data could yield further insight into whether the mechanisms we describe are sufficient for coexistence of the two species over time.

We faced several logistical constraints in our study that limited our ability to parse dog–fox interactions in Spiti. The rugged terrain and harsh winter severely limited our ability to travel between villages during inclement weather. This limited our ability to sample the dog-density gradient more continuously. The clumped nature of villages within each dog category means that we could not account for any localized effects of the sites chosen that may have impacted our responses or introduced noise. Follow-up studies would do well to include more villages in each dog category if logistics allow and explicitly including site-level effects in our analyses. The ability to determine sex and identify individual foxes would have given us a more nuanced understanding of fox vigilance across the dog-density gradient. The fear responses of females, which must take care of offspring and often hold stable territories, are different from those of males [80]. Since our experiment was measuring risk perception and responses, incorporating data about the body condition or age of individual foxes would have yielded more robust insights. Animals that are starved or have poor body condition may engage in riskier behaviour to obtain resources [18,81]. Thus, the way foxes respond to simulated dog presence may differ, even within the same individual. Additionally, stratifying the responses by age category may have yielded insights into how and when foxes use reactive vigilance in Spiti. Responses can also vary based on individual personalities of foxes, with some individuals being bolder than others [82]. Additionally, while we tried to place foraging stations in flat areas, such as pastures, we did not explicitly account for variations in spatial complexity, which can affect risk perception [42]. A methodological drawback in our foraging station experiment was the use of just a single exemplar of barks and moos. The use of multiple exemplars of sounds prevents the habituation of foxes through exposure [44,54]. We attempted to minimize habituation by repeating each treatment only once every 7 days. We also baited our foraging stations in the afternoon hours (between 14.00 and 17.00). While this was done to prevent consumption of bait by other animals, it meant that most of our first-visit vigilance responses were captured before midnight. Fox responses towards dog cues may change over a night, as the probability of encountering a dog reduces at night and rises in the day. Incorporating such finer-scale diel variations into our experiment may have yielded further insight into the context of vigilance behaviour by foxes in Spiti.

Our findings highlight the need to investigate fine-scale interactions between carnivores to better inform conservation outcomes. Fine‐scale interactions among carnivores have increasingly been recognized as key to understanding how species partition space and time to mitigate direct conflict [83]. For example, in Tanzania, fine-scale interactions were key to coexistence of lions, leopard and wild dogs over a large, mixed-use landscape [83]. Similarly, bobcats, coyotes and cougars in the USA show highly overlapping niches, but coexist through fine-scale avoidance, minimizing direct encounters [84]. As suitable habitats reduce, and more carnivores share spaces and home ranges, investigations into such fine-scale mechanisms of coexistence are crucial to conservation and management efforts. Further research into the fox-dog paradigm in Spiti may benefit from the incorporation of telemetry, enabling spatiotemporal tracking of the two canids at finer scales. Such data may shed light on whether the vigilance responses we report, translate to actual fine-scale avoidance of dogs by foxes. Additionally, such fine-scale data on dog movements may yield important insights for carnivore conservation in Spiti. Dogs in Spiti show high seroprevalence for viral pathogens like canine distemper and canine parvovirus [85]. Understanding how carnivores move and navigate this shared landscape may provide insights into possible disease transmission between dogs and the wider carnivore community.

While we show that foxes can mitigate the risk of encountering and having conflict with dogs and persist at higher relative abundances in areas with more dogs, the negative effects of the latter on foxes and wildlife as predators, competitors and disease reservoirs cannot be ignored [12,13]. It must also be noted that the red fox is a highly adaptive, generalist mesopredator, with near-global distributions due to its behavioural plasticity and ability to thrive among anthropogenic disturbances [86]. Regions in the global south, like South Asia, South America, Oceania and Africa, which host diverse mesopredator communities, also host the largest free-ranging dog populations [1,13]. In the face of increasing human footprints, it is crucial to investigate and understand how the predator guild, especially specialist mesocarnivores, cope with the spread of dogs.

## Data Availability

All our data, associated metadata and associated R scripts can be found on Dryad [87]. Supplementary material is available online [88].
